# Serum LINC01127 serves as a diagnostic biomarker for sepsis and its predictive value for clinical outcomes

**DOI:** 10.1186/s41065-026-00670-1

**Published:** 2026-04-06

**Authors:** Congcong Li, Xiao Gui, Haiyuan Zhu, Jinhui Wang, Yu Xie, Lilin Wang, Yongyang Tian, Shun Li

**Affiliations:** 1 Department of Critical Care Medicine, Sir Run Run Shaw Hospital, School of Medicine, Zhejiang University, Hangzhou, 310016 China; 2 Department of Critical Care Medicine, University-Town Hospital of Chongqing Medical University, Chongqing, 401331 China; 3 Department of Respiratory and Critical Care Medicine, University-Town Hospital of Chongqing Medical University, Chongqing, 401331 China; 4 Department of Critical Care Medicine, The Affiliated Hospital of Xuzhou Medical University, No.99, Huaihai Road, Xuzhou, 221000 China

**Keywords:** Sepsis, LINC01127, miR-34b-5p, Diagnosis, Prognosis

## Abstract

**Background:**

This study aims to investigate the role of LINC01127 in sepsis and evaluate its potential clinical value.

**Methods:**

This study included 102 sepsis patients and 102 controls matched for age and gender. Serum levels of LINC01127 and miR-34b-5p were measured using quantitative real-time PCR (RT-qPCR). Receiver operating characteristic (ROC) curves were constructed to assess the diagnostic efficacy of LINC01127 for sepsis, while Kaplan-Meier curves evaluated its prognostic significance during 28-day follow-up. Lipopolysaccharide (LPS)-induced THP-1 cells were used to simulate an in vitro sepsis model. Enzyme-linked immunosorbent assay (ELISA) measured proinflammatory factor levels, and flow cytometry assessed apoptosis.

**Results:**

Serum LINC01127 exhibited downregulation in sepsis-affected individuals, while miR-34b-5p was upregulated. ROC curve analysis demonstrated that LINC01127 possesses good diagnostic value for sepsis. Kaplan-Meier analysis showed that low LINC01127 levels were associated with poorer prognosis during the 28-day follow-up. Bioinformatics analysis successfully predicted a binding site for miR-34b-5p on LINC01127, and dual-luciferase reporter (DLR) and RNA immunoprecipitation (RIP) assays confirmed their target relationship. Cell experiments demonstrated that upregulating LINC01127 significantly reduced the apoptosis and inflammation triggered by LPS, while increasing miR-34b-5p expression markedly reversed these effects.

**Conclusion:**

LINC01127 may serve as a promising biomarker for sepsis, alleviating apoptosis and inflammatory responses through targeting miR-34b-5p. This offers novel insights into sepsis diagnosis and treatment.

**Supplementary Information:**

The online version contains supplementary material available at https://doi.org/10.1186/s41065-026-00670-1.

## Background

Sepsis, triggered by infection or trauma, can lead to organ failure and even shock in severe cases. It is the primary cause of death in intensive care unit [1]. It is estimated that approximately 31.5 million people worldwide develop sepsis annually, with over 5 million potential deaths [2–4]. The 28-day mortality rate is approximately 20% [5]. Currently, biomarkers used for sepsis diagnosis exhibit limited specificity and sensitivity [6]. Furthermore, accurate prediction tools remain scarce, leading to clinical treatment delays. Therefore, identifying novel biomarkers for the early diagnosis and prognosis assessment of sepsis is crucial for improving patient management and clinical outcomes.

Recent studies have revealed that dysregulation of lncRNA correlates with various pathological processes [7]. The onset and progression of sepsis are also closely linked to abnormal lncRNA expression [8, 9]. Among these, LINC01127 has been reported to be dysregulated in multiple inflammatory and organ injury. For instance, it has been suggested as a potential diagnostic marker for lupus nephritis [10] and may play a role in muscle injury and heart failure [11]. Furthermore, an RNA sequencing study identified differential expression of LINC01127 in sepsis patients [12], though its specific mechanism of action in sepsis remains unelucidated.

MicroRNAs (miRNAs) play significant roles in the pathogenesis of various diseases [13–15]. Multiple lines of evidence suggest that a connection involving miR-34b-5p and inflammation. One study revealed that miR-34b-5p expression is upregulated in sepsis-initiated acute kidney injury and correlates with enhanced inflammatory responses [16]. Another investigation using a sepsis-induced acute lung injury model demonstrated that inhibiting miR-34b-5p alleviates pulmonary inflammation [17]. Furthermore, lncRNA can alleviate sepsis-induced myocardial injury and inflammatory responses by binding to miR-34b-5p and modulating downstream signaling pathways [18, 19]. Bioinformatics analysis suggests LINC01127 and miR-34b-5p may have potential binding sites. However, the specific molecular mechanisms underlying their interaction in sepsis remain unexplored.

Therefore, this study aims to investigate LINC01127 expression level in sepsis patients, evaluate its clinical value, and explore its regulatory relationship with miR-34b-5p. This findings may provide novel molecular biomarkers and a theoretical foundation for the early diagnosis and targeted treatment of sepsis.

## Materials and methods

### Participants and clinical data collection

This study included 102 controls and 102 sepsis patients from the ICU of University-Town Hospital of Chongqing Medical University between October 2021 and December 2024. Inclusion criteria: patients meeting the diagnostic criteria for sepsis and septic shock defined by the 2016 Third International Consensus; time from diagnosis to ICU admission within 48 h. Exclusions included patients with cancer, chronic inflammation, severe immunodeficiency, prior immunotherapy before admission, or those in pregnancy or lactation. All study protocols were approved by the Ethics Committee of University-Town Hospital of Chongqing Medical University. After obtaining written informed consent, 5 mL peripheral venous blood was collected from all participants. For patients with sepsis, blood samples were collected within 48 h of ICU admission. For controls, blood was obtained during a health assessment visit. Blood samples were centrifuged to collect serum, which was immediately transferred to -80 °C freezers for storage. Clinical information for all participants was documented.

The sample size of this study was estimated through the Gpower software for power analysis. Under the conditions of setting the significance level at 0.05, the effect size at 0.3, and the test power at 80%, the calculated minimum sample size required was 180 cases. Eventually, a total of 204 subjects were included, which met and exceeded the estimated minimum sample size requirement, ensuring the statistical power of the study.

### Cell culture and sepsis model establishment

THP-1 cells (ATCC, USA) were cultured in RPMI-1640 medium (Gibco, USA) containing 10% FBS (Gibco, USA) and 1% penicillin/streptomycin (Invitrogen, USA) at 37 °C with 5% carbon dioxide. To build a sepsis model, cells were exposed to 1 µg/mL lipopolysaccharide (LPS, Sigma Aldrich, USA) at 37 °C for 24 h.

### Cell transfection

Plasmids for overexpressing LINC01127 (oe-LINC01127#1 and oe-LINC01127#2) and oe-NC were synthesized by Shanghai GenePharma. Concurrently, miR-34b-5p mimics and mimic NC were provided by RiboBio in Guangzhou. Following LPS treatment, THP-1 cells were transfected by Lipofectamine^®^ 3000 transfection reagent (Invitrogen, USA). 48 h after cell transfection, transfection efficiency was assessed via *reverse transcription-quantitative polymerase chain reaction (*RT-qPCR).

### RT-qPCR

RNA was extracted from THP-1 cells and serum using TRIzol reagent (Invitrogen, USA). Its concentration and purity were assessed on a NanoDrop 2000 spectrophotometer. Take RNA and reverse transcribe lncRNA or miRNA into cDNA through the PrimeScript RT Kit (Takara, Dalian) or TaqMan miRNA Reverse Transcription Reagent (Life Technologies, USA), respectively, and following the SYBR Green protocol. LINC01127/miR-34b-5p were quantified by the 2^−ΔΔCt^ method, normalized against GPAHD/U6 as internal control genes, respectively.

### Enzyme-linked immunosorbent assay (ELISA)

Inflammatory cytokine concentrations were evaluated by ELISA kit (Invitrogen, USA), including interleukin-6 (IL-6, Cat# KRC0061), tumor necrosis factor-α (TNF-α, Cat# KAC1751), and interleukin-1β (IL-1β, Cat# KAC1211). All procedures were strictly followed the kit instructions. The absorbance values at 450 nm were read using an ELISA reader.

### Flow cytometry

In 24-well plates, each well received 1 × 10⁵ cells for seeding. After 48 h of culture, cells were harvested, resuspended in 100 µL binding buffer, and incubated with 5 µL PI and 5 µL Annexin V-FITC (Beyotime, Shanghai) staining solution for 10 min in the dark. Subsequently, A BD/FACS Aria II flow cytometer (BD Biosciences, USA) utilized for the analysis of stained cells (BD Biosciences, USA).

### Subcellular fractionation

THP-1 cells were lysed for 10 min using pre-chilled cell fractionation buffer, followed by separation using the PARIS assay kit (Thermo Fisher Scientific, USA). RNA was subsequently isolated, and the LINC01127 was evaluated through RT-qPCR. Cytoplasmic and nuclear fractions were normalized to GAPDH/U6 as internal controls, respectively.

### Dual-luciferase reporter (DLR) assay

Using the lncRNASNP2 (https://guolab.wchscu.cn/lncRNASNP/#!) and TargetScan 8.0 (https://www.targetscan.org/vert_80/) database, we successfully predicted a potential binding site between LINC01127 and miR-34b-5p, as well as between the 3’UTR of apolipoprotein B (APOB) and miR-34b-5p. Based on these predictions, we constructed wild-type (WT) and mutant (MUT) plasmids for LINC01127 and APOB, which were cloned into the pGL3 luciferase reporter vector (Promega, USA). THP-1 cells were seeded in 24-well plates. Using Lipofectamine 3000 transfection reagent, the plasmids were co-transfected into cells with miR-34b-5p mimics, miR-34b-5p inhibitors, negative control mimics, and negative control inhibitors, respectively. After 48 h, luciferase activity was assessed by a DLR assay system.

### RNA immunoprecipitation (RIP) assay

RIP experiments were performed using the EZ-MAGNARIP Kit (Cat#17–700, Millipore, USA). After treating cell samples with RIP lysis buffer, the lysates were incubated overnight at 4 °C on a shaking incubator with magnetic beads coated with anti-Ago2 or anti-IgG antibodies. The LINC01127, miR-34b-5p and APOB levels were detected by RT-qPCR in the precipitates.

### Data analysis

Experimental data were analyzed by SPSS 22.0 and GraphPad Prism 9.0 and represented by mean ± SD. The normality of the data was evaluated through the Kolmogorov-Smirnov test. Normal distribution data were compared between two groups using t-tests, or among multiple groups using two-factor analysis of variance. If there was a significant interaction, a simple effect analysis was conducted based on Bonferroni correction. For one-way multiple group comparisons, analysis of variance was first used, and if the results were significant, Tukey’s HSD post-hoc test was performed. For non-normal distribution data, the Mann-Whitney U test was used for comparison. Categorical variables were expressed as frequencies and proportions, and comparisons between groups were conducted using the chi-square test. Survival analysis was performed using Kaplan–Meier method with log‑rank test, and Cox proportional hazards regression was used to evaluate prognostic factors. Statistical significance was defined as a *P* value below 0.05.

## Results

### Analysis of baseline characteristics of study participants

This study first compared baseline clinical data between 102 enrolled sepsis patients and 102 controls. The two groups did not differ markedly in age, gender, BMI, or smoking history (*P* > 0.05). However, compared with controls, sepsis patients exhibited significantly elevated levels of SCR, white blood cells, CRP, PCT, TNF-α, IL-1β, IL-6, and IL-8 at enrollment, while albumin levels were markedly decreased (*P* < 0.0001, Table 1). These results confirm that the sepsis patients enrolled in the present study are clinically representative.

**Table 1:** Comparison between sepsis patients and health controls

Indicators	Controls (*n* = 102)	Sepsis patients (*n* = 102)	*P*
Age, years	56.06 ± 10.77	57.34 ± 10.25	0.384^a^
Gender, male/female	50/52	60/42	0.160^b^
BMI, kg/m^2^	24.21 ± 4.33	24.66 ± 4.06	0.447^a^
Smoke, %	43.14	47.06	0.574^b^
SCR, mg/dL	0.97 ± 0.29	1.69 ± 0.29	**0.000** ^a^
Albumin, g/L	39.91 ± 3.32	27.24 ± 3.84	**0.000** ^a^
WBC, ×10^9^/L	7.52 ± 1.77	13.51 ± 7.39	**0.000** ^a^
CRP, mg/L	4.16 ± 1.17	95.79 ± 29.38	**0.000** ^a^
PCT, mg/L	5.11 ± 1.54	11.92 ± 3.69	**0.000** ^a^
IL-6, pg/mL	13.01 ± 1.71	51.58 ± 3.71	**0.000** ^a^
TNF-α, pg/mL	30.06 ± 2.60	199.23 ± 34.12	**0.000** ^a^
IL-1β, pg/mL	25.87 ± 2.64	180.74 ± 27.28	**0.000** ^a^
SOFA score	-	7.78 ± 2.37	-
APACHE II score	-	15.07 ± 4.37	-
Death, n	-	28	-

*BMI* body mass index, *SCR* serum creatinine, *WBC* white blood cells, *CRP* C-reactive protein, *PCT* procalcitonin, *IL* Interleukin, *TNF* Tumor necrosis factor, *SOFA* sequential organ failure assessment, *APACHE II* acute pathology and chronic health evaluation II

*P*^a^ independent sample t-test,  *P*^b^ Chi-square test was used. Statistical significance was set at *P*< 0.05, and significant results are highlighted in bold

### Expression of LINC01127 in sepsis’ serum and its clinical value

**Fig. 1:**
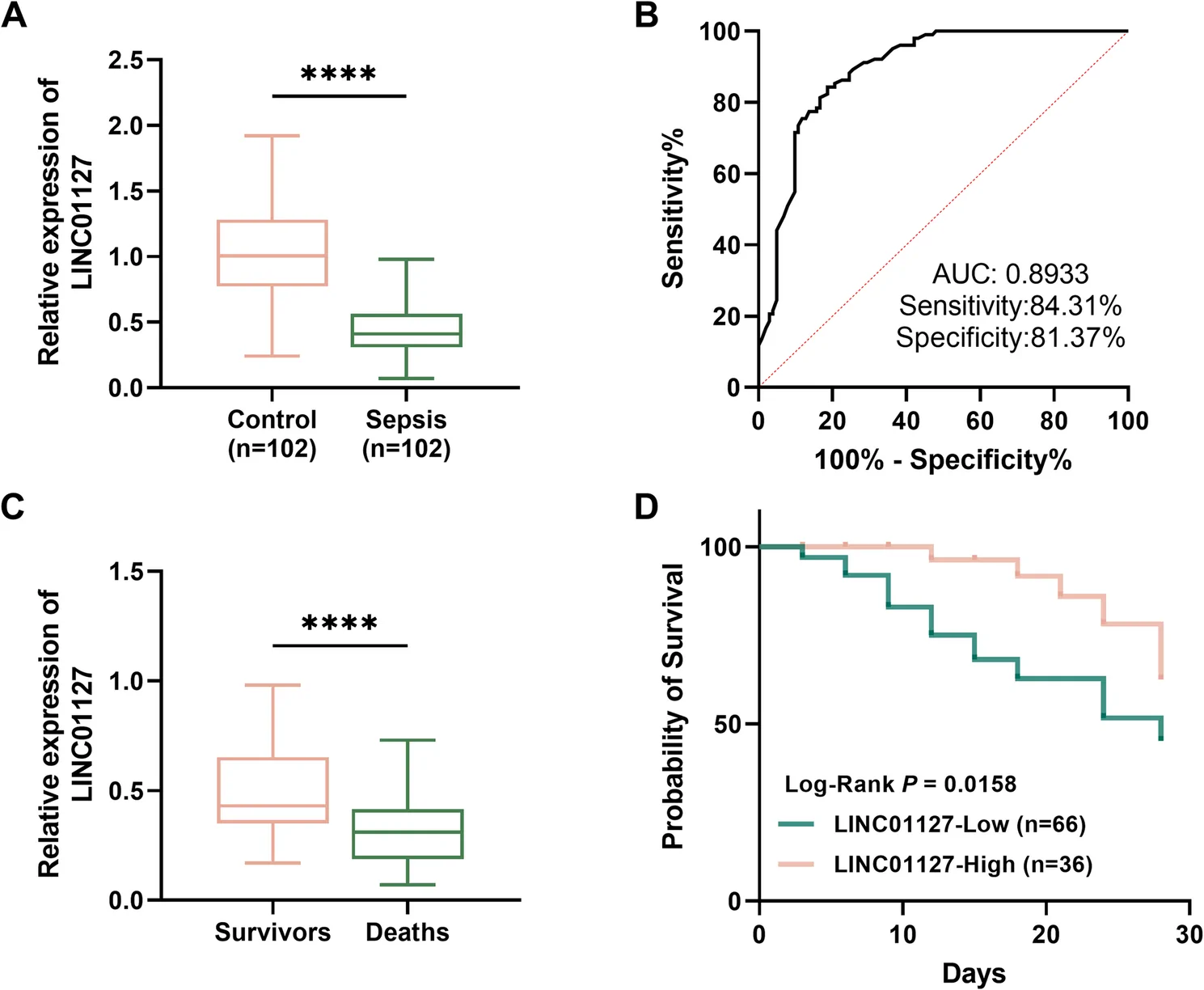
Abnormal downregulation of LINC01127 in serum of sepsis patients and its diagnostic and prognostic potential for sepsis. **A** LINC01127 expression is significantly downregulated in serum from sepsis patients. **B** ROC curve analysis indicates serum LINC01127 effectively distinguishes sepsis patients from controls. **C **After 28-day follow-up, serum LINC01127 levels in survivors were significantly higher than in non-survivors. **D** Kaplan-Meier curve shows poorer prognosis in the low-expression group. **** *P* < 0.0001

We measured LINC01127 levels in the subjects’ serum. Results showed that LINC01127 expression was significantly lower in sepsis patients than in controls (*P* < 0.0001, Fig. 1A). Furthermore, analysis of the correlation between LINC01127 levels and key indicators in sepsis patients revealed that LINC01127 levels were negatively correlated with CRP (*r* = − 0.458, *P* < 0.0001), PCT (*r* = − 0.558, *P* < 0.0001), IL-6 (*r* = − 0.653, *P* < 0.0001), TNF-α (*r* = − 0.539, *P* < 0.0001) and IL-1β (*r* = − 0.588, *P* < 0.0001). Additionally, negative correlations were observed with disease severity scores SOFA (*r* = − 0.571, *P* < 0.0001) and APACHE II (*r* = − 0.609, *P* < 0.0001) (Table 2). All of the correlations remained statistically significant after multiple corrections. ROC curve analysis demonstrated that LINC01127 effectively distinguished sepsis patients from controls, with an AUC of 0.8933 and sensitivities and specificities of 84.31% and 81.37%, respectively (*P* < 0.0001, Fig. 1B). Furthermore, 5-fold internal cross-validation was performed to evaluate the reliability of the ROC curve analysis, and the results verified the robustness of the above findings (Supplementary Figure S1). Furthermore, a 28-day follow-up of sepsis patients revealed markedly increased LINC01127 in survivors in comparison with non-survivors (*P* < 0.0001, Fig. 1C). Further stratification of 102 patients into high-expression (*n* = 36) and low-expression (*n* = 66) groups with the cut-off value set as the mean expression level of LINC01127 revealed poorer prognosis in low-expression (*P* = 0.0158, Fig. 1D). To verify the robustness of the grouping method, we re-grouped according to the median expression level of LINC01127. The Kaplan-Meier analysis showed that the 28-day survival rate of the low-expression group was still significantly lower than that of the high-expression group (*P* = 0.0178, Supplementary Figure S2). Cox regression analysis showed that, even after adjusting for all confounding factors, LINC01127 was still associated with a higher risk of death within 28 days in sepsis (*P* = 0.012, Table 3).

**Table 2:** Correlation between LINC01127 levels and clinical indicators in sepsis patients

Indicators	LINC01127	
	*r*	*P*
Age, years	-0.085	0.398
Gender, male/female	-0.102	0.307
BMI, kg/m2	0.044	0.661
Smoke, %	-0.103	0.304
SCR, mg/dL	-0.215	0.03
Albumin, g/L	0.214	0.03
WBC, ×10^9^/L	-0.288	0.004
CRP, mg/L	-0.458	**0.000**
PCT, mg/L	-0.558	**0.000**
IL-6, pg/mL	-0.653	**0.000**
TNF-α, pg/mL	-0.539	**0.000**
IL-1β, pg/mL	-0.588	**0.000**
SOFA score	-0.571	**0.000**
APACHE II score	-0.609	**0.000**

*SCR* serum creatinine, *WBC* white blood cells, *CRP* C-reactive protein, *PCT* procalcitonin, *IL* Interleukin, *TNF* Tumor necrosis factor, *SOFA* sequential organ failure assessment, *APACHE II* acute pathology and chronic health evaluation II. Statistical significance was set at *P*< 0.05, and significant results are highlighted in bold

**Table 3:** Univariate and multivariate Cox analysis of LINC01127 and clinical parameters with overall survival

Indicators	Univariate analysis				Multivariate analysis			
	*P*	OR	95% CI	*P*		OR		95% CI
Age, years	0.822	1.089	0.519–2.287					
Gender, male/female	0.396	1.412	0.650–3.067					
BMI, kg/m^2^	0.239	1.578	0.738–3.372				0	
Smoke, %	0.138	1.795	0.828–3.892					
SCR, mg/dL	0.416	1.365	0.645–2.886					
Albumin, g/L	0.243	0.637	0.299–1.359					
WBC, ×10^9^/L	0.073	2.036	0.935–4.434					
CRP, mg/L	0.135	1.791	0.834–3.847	0.261		0.625	0.275–1.420	
PCT, mg/L	0.000	13.152	3.898–44.372	0.278		2.388	0.495–11.523	
IL-6, pg/mL	0.000	8.517	3.166–22.914	0.409		1.876	0.422–8.347	
TNF-α, pg/mL	0.000	6.732	2.746–16.506	0.210		2.121	0.655–6.866	
IL-1β, pg/mL	0.000	8.577	2.962–24.834	0.353		2.157	0.426–10.909	
SOFA score	0.000	9.499	3.412–26.441	0.038		5.338	1.094–26.046	
APACHE II score	0.000	7.979	3.199–19.899	0.017		5.315	1.347–20.962	
LINC01127	0.025	0.331	0.126–0.873	0.016		0.141	0.029–0.696	

*SCR* serum creatinine *WBC* white blood cells, *CRP* C-reactive protein, *PCT* procalcitonin, *IL* Interleukin, *TNF* Tumor necrosis factor, *SOFA* sequential organ failure assessment, *APACHE II* acute pathology and chronic health evaluation II

### Overexpression of LINC01127 reverses LPS-induced inflammation and apoptosis

**Fig. 2:**
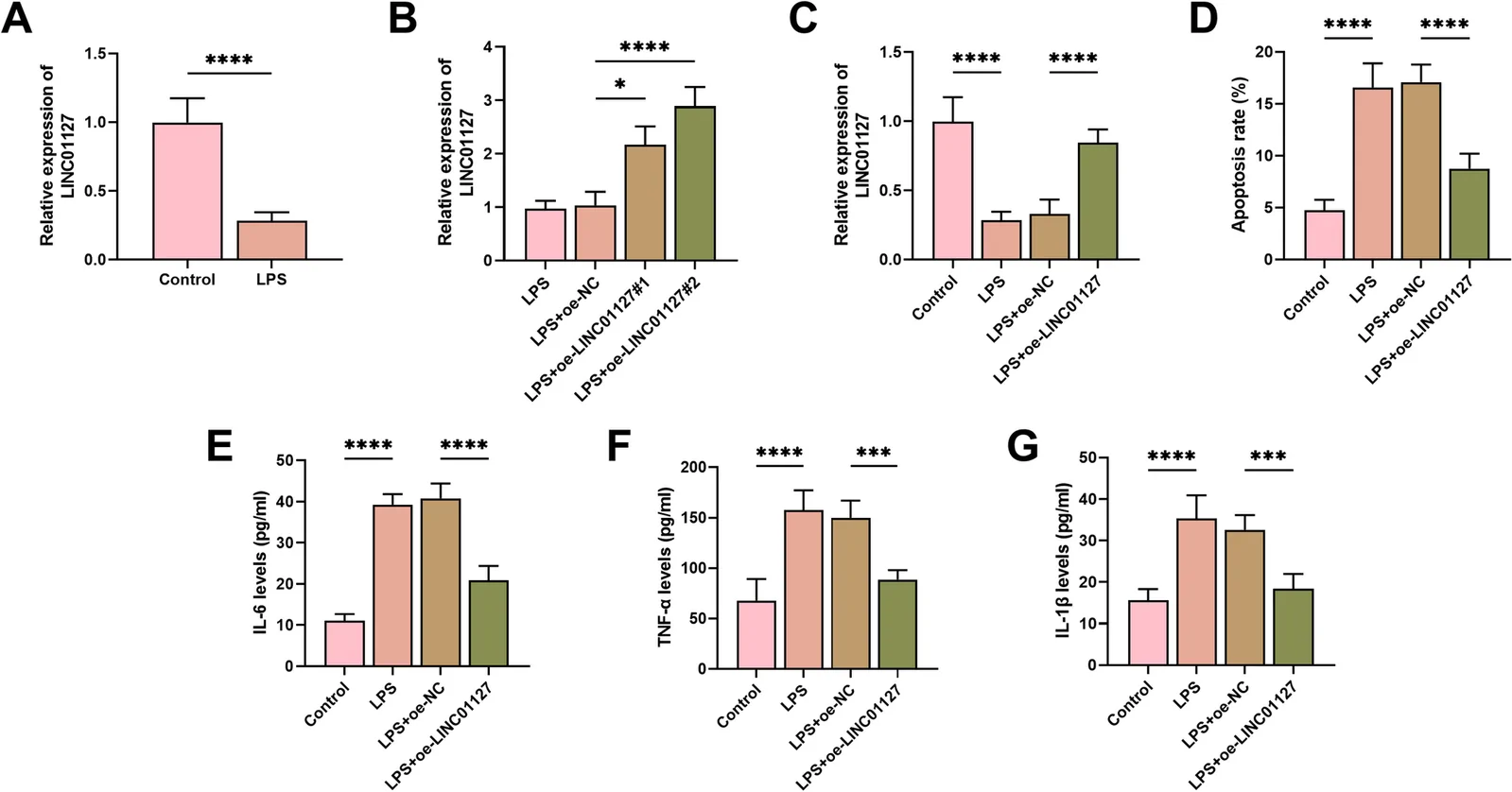
Upregulation of LINC01127 alleviates LPS-induced inflammation and apoptosis in THP-1 cells. **A** Detection of LINC01127 expression in LPS-treated THP-1 cells via RT-qPCR. **B** Assessment of LINC01127 overexpression efficiency via RT-qPCR. **C**. RT-qPCR analysis of the effect of oe-LINC01127 transfection on LINC01127 expression following LPS treatment. **D** Flow cytometry assessment of the effect of LINC01127 upregulation on apoptosis. ELISA analysis of the effect of LINC01127 upregulation on inflammatory cytokine IL-6 (**E**), TNF-α (**F**), and IL-1β (**G**) expression. * *P* < 0.05, *** *P* < 0.001, **** *P* < 0.0001

Figure 2A shows that LPS stimulation markedly reduced LINC01127 expression in THP-1 cells compared with the control group (*P* < 0.0001). We then transfected two LINC01127 overexpression plasmids (oe-LINC01127#1 and oe-LINC01127#2) into THP-1 cells, and oe-LINC01127#2 demonstrated higher upregulation efficiency (*P* < 0.05, Fig. 2B). Therefore, oe-LINC01127#2 was used in subsequent experiments and designated as oe-LINC01127. Overexpression of LINC01127 significantly reversed the LPS-induced decrease in LINC01127 expression (*P* < 0.0001, Fig. 2C). Concurrently, LPS-induced apoptotic increase was markedly attenuated upon LINC01127 overexpression (*P* < 0.0001, Fig. 2D). Upregulating LINC01127 also significantly suppressed the enhanced secretion of proinflammatory cytokines in LPS-stimulated THP-1 cells (*P* < 0.001, Fig. 2E-G).

### LINC01127 acts as a sponge for miR-34b-5p

**Fig. 3:**
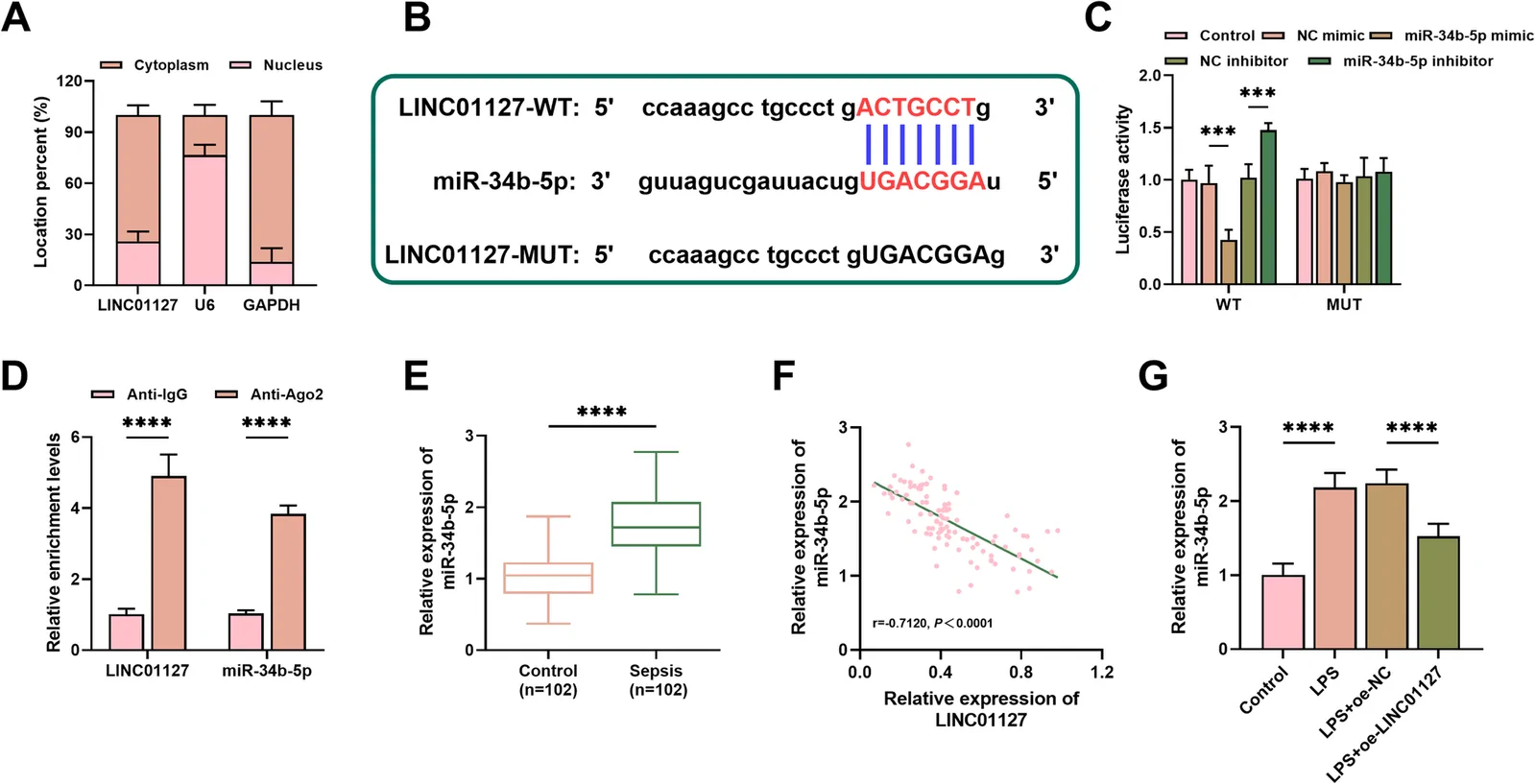
miR-34b-5p is a downstream effector of LINC01127. **A** Subcellular localization analysis shows LINC01127 localized to the cytoplasm. **B** LINC01127 and miR-34b-5p binding sites predicted by lncRNA-SNP2, used to construct LINC01127-WT/MUT reporter plasmids. **C**-**D** DLR and RIP experiments confirm LINC01127 targets miR-34b-5p. **E** RT-qPCR detection of miR-34b-5p expression in serum from sepsis patients. **F** Pearson correlation analysis of the relationship between LINC01127 and miR-34b-5p in serum from sepsis patients. **G** RT-qPCR detection of the effect of oe-LINC01127 transfection on miR-34b-5p expression. *** *P* < 0.001, **** *P* < 0.0001

Figure 3A shows that LINC01127 is primarily localized in the cytoplasm of THP-1 cells. Using the lncRNASNP2 website, we successfully predicted potential binding sites between LINC01127 and miR-34b-5p, and constructed LINC01127-WT/MUT reporter plasmids based on these predictions (Fig. 3B). DLR results demonstrated that the miR-34b-5p mimic significantly reduced the luciferase activity of LINC01127-WT, whereas its inhibitor markedly enhanced it. Neither treatment significantly affected the fluorescence activity of LINC01127-MUT (*P* < 0.001, Fig. 3C). Compared to the Anti-IgG group, LINC01127 and miR-34b-5p were markedly enriched in Anti-Ago2 group (*P* < 0.0001, Fig. 3D). Furthermore, serum miR-34b-5p expression was significantly upregulated in sepsis patients compared to controls (*P* < 0.0001, Fig. 3E) and showed a significant negative correlation with LINC01127 expression (*r* = -0.712, *P* < 0.0001, Fig. 3F). In LPS-induced THP-1 cells, miR-34b-5p expression was significantly elevated, but upregulation of LINC01127 markedly reversed this upregulation (*P* < 0.0001, Fig. 3G).

### *LINC01127 regulates LPS-triggered injury in THP-1 cells by targeting miR-34b-5p*

**Fig. 4:**
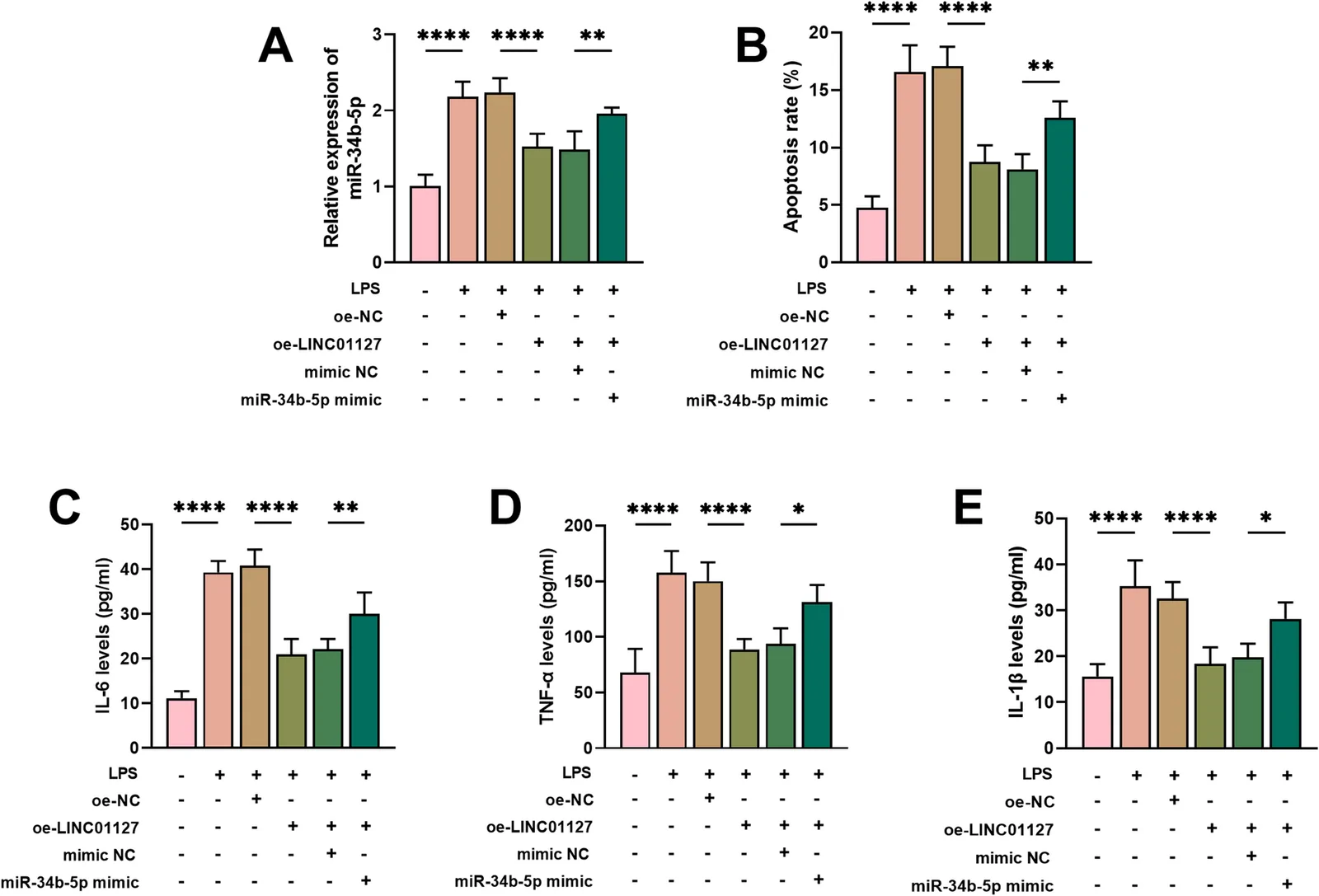
LINC01127 influences LPS-induced damage to THP-1 cells by targeting miR-34b-5p. **A** RT-qPCR analysis of the effects of different transfection treatments on miR-34b-5p expression. **B** Flow cytometry assessment of the effects of different transfection treatments on apoptosis. ELISA evaluation of the effects of different transfection treatments on inflammatory cytokinesIL-6 (**C**), TNF-α (**D**), and IL-1β (**E**). * *P* < 0.05, ** *P* < 0.01, **** *P* < 0.0001

To investigate the impact of LINC01127 and miR-34b-5p on LPS-induced cellular damage, we first assessed the miR-34b-5p following transfection with oe-LINC01127. LPS treatment significantly reduced miR-34b-5p levels upon LINC01127 overexpression, however, miR-34b-5p mimic markedly reversed this trend (*P* < 0.01, Fig. 4A). Increasing miR-34b-5p markedly attenuated the anti-apoptotic influence mediated by LINC01127 upregulation following LPS treatment (*P* < 0.01, Fig. 4B). Concurrently, increasing miR-34b-5p markedly suppressed the reduction in inflammatory cytokine release from THP-1 cells induced by LINC01127 overexpression (*P* < 0.05, Fig. 4C-E).

### APOB is a downstream target of miR-34b-5p

**Fig. 5:**
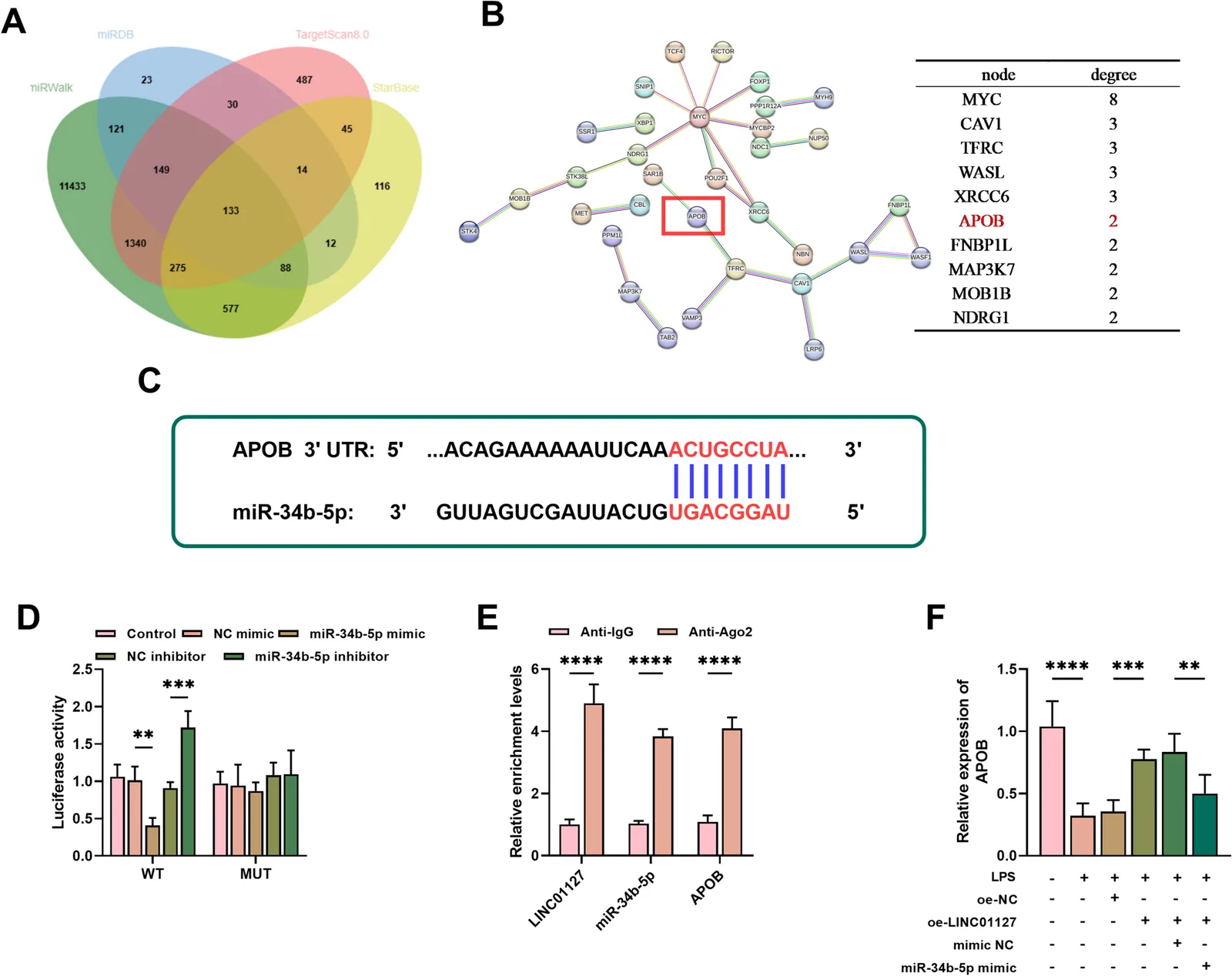
APOB is a direct target gene of miR-34b-5p. **A** Venn diagram showing the intersection of miR-34b-5p target genes predicted by TargetScan, miRDB, miRWalk, and StarBase databases. **B** Protein-protein interaction network analysis of overlapping target genes. **C** TargetScan predicts potential binding sites between miR-34b-5p and the APOB 3’UTR region. **C**-**D** DLR assay and RIP assay validate the target binding between miR-34b-5p and APOB. **E** Changes in APOB expression levels in THP-1 cells under different transfection conditions. * *P* < 0.05, ** *P* < 0.01, **** *P* < 0.0001

Figure 5A shows the results of miR-34b-5p target prediction using the TargetScan, miRDB, miRWalk, and StarBase databases. PPI network analysis of overlapping target genes identified APOB as a highly credible target of miR-34b-5p (*P* = 0.0423, Fig. 5B). Subsequently, potential binding sites between miR-34b-5p and the APOB 3’ UTR region were predicted (Fig. 5C). To validate the target relationship, a DLR assay was first performed. Results showed that miR-34b-5p mimic significantly inhibited luciferase activity in APOB-WT, while the miR-34b-5p inhibitor significantly enhanced its activity; neither had a significant effect on APOB-MUT (*P* < 0.01, Fig. 5D). A RIP assay further confirmed that LINC01127, miR-34b-5p, and APOB were significantly enriched in the Anti-Ago2 (*P* < 0.0001, Fig. 5E). In LPS-treated THP-1 cells, APOB expression was markedly downregulated; overexpression of LINC01127 restored APOB levels, while transfection with miR-34b-5p mimics effectively reversed this upregulation (*P* < 0.01, Fig. 5F).

## Discussion

Differentiating sepsis from immune disorders remains a significant clinical challenge [20]. Although traditional biomarkers (e.g., CRP, PCT) and clinical scoring systems are widely used in the diagnosis of sepsis, their diagnostic accuracy is limited [21–24]. Therefore, developing novel biomarkers with high sensitivity and specificity is essential to enable early identification and precise intervention for sepsis, thereby improving patient outcomes. The present study explored the diagnostic and predictive value of LINC01127 in sepsis and its effect on the disease. LINC01127 was markedly decreased in sepsis serum, negatively correlated with disease severity scores and proinflammatory factors, and served as an effective marker for distinguishing sepsis patients from controls and predicting adverse patient outcomes. LINC01127 may function as a molecular sponge for miR-34b-5p, thereby modulating the release of inflammatory cytokines and influencing apoptotic processes.

ncRNAs have been recognized as important biomarkers due to their stability in body fluids and differential expression in diseases [25–27]. Our study found that LINC01127 levels were significantly reduced in sepsis serum and negatively correlated with commonly used clinical diagnostic biomarkers and disease severity scores (SOFA and APACHE II) [28]. ROC analysis further validated the excellent diagnostic efficacy of serum LINC01127 for sepsis, Notably, multivariate analysis results from our study demonstrated that the predictive value of LINC01127 is independent of the currently clinically used severity scores and biomarkers, which suggests that it may reflect disease dimensions not fully captured by existing indicators and thus possesses potential incremental prognostic value. A subsequent 28-day follow-up revealed that patients with low LINC01127 expression had significantly poorer clinical outcomes, which collectively demonstrate the potential of LINC01127 as a novel biomarker for sepsis diagnosis and prognostic stratification. Certain lncRNAs participate in modulating sepsis progression by controlling inflammatory factors such as IL-6, IL-1β, and TNF-α [29]. For example, lncRNA MCM3AP-AS1 has been implicated in sepsis-associated inflammatory processes [30]. In septic patients, lncRNA UCA1 correlates with the release of multiple proinflammatory cytokines and poor prognosis [31]. Recently identified LINC01127 exhibits abnormal expression in ovarian cancer [32] and visceral leishmaniasis [33] and may be associated with poor prognosis in glioblastoma patients [34]. Furthermore, LINC01127 has been found to be abnormally expressed in active lupus nephritis, implying its conserved regulatory role in inflammatory processes across immune-related disorders [10], which further supports its functional relevance in sepsis-associated inflammation. The lncRNAs biological functions are often associated with their subcellular localization [35]. This study first confirmed that LINC01127 localizes to the cytoplasm. To further investigate its role in sepsis, we employed an LPS-induced cellular model. Results showed that upregulating LINC01127 significantly reversed LPS-induced apoptosis and inflammation.

Through bioinformatics prediction and experimental validation, we confirmed miR-34b-5p as a functional target of LINC01127. Previous studies have shown that miR-34b-5p is involved in various inflammatory processes. For instance, it contributes to the progression of colitis [36], modulates the inflammatory response in liver disease [37], and influences inflammation and oxidative damage in renal tubular epithelial cells [38]. Furthermore, we observed abnormal expression of miR-34b-5p in periodontitis induced by Streptococcus gordonii dental infection [39]. miR-34b-5p overexpression exacerbates sepsis-induced organ injury [16, 40]. Consistent with these findings, our study revealed elevated levels of miR-34b-5p in sepsis, which were negatively correlated with LINC01127 expression. Rescue experiments further demonstrated that increased miR-34b-5p miR-34b-5p significantly reversed the inhibitory effect of LINC01127 upregulation on LPS-induced cellular inflammation and apoptosis.

To further clarify the downstream pathway, we conducted bioinformatics analysis (TargetScan, miRDB, miRWalk, StarBase) to predict that APOB is a highly reliable target of miR-34b-5p. It has been reported that in sepsis, the decrease in serum APOB levels is associated with aggravated inflammatory response and severity of the disease [41, 42]. Additionally, APOB is associated with organ dysfunction in sepsis patients [43], and its genetic variations may affect the 28-day mortality rate of patients [44]. This study further confirms that miR-34b-5p directly targets and suppresses APOB expression. Based on this, we propose the scientific hypothesis that LINC01127 may act as an endogenous competitive RNA to sequester miR-34b-5p, thereby relieving its inhibitory effect on the target gene APOB and forming the LINC01127/miR-34b-5p/APOB regulatory axis. Subsequent work will focus on validating the regulatory mechanisms and downstream functions of these three components around this hypothesis, aiming to systematically elucidate the critical role of this pathway in the pathogenesis and progression of sepsis.

This study still has several limitations. Firstly, this study is a single-center, exploratory study. The model performance is mainly based on the development cohort and lacks independent external validation, and the sample size is limited. Although strict inclusion and statistical correction have been carried out, the results may still be affected by regional population characteristics and clinical practice. Additionally, some important clinical confounding factors such as patient comorbidities were not included in the analysis, which may to some extent limit the clinical applicability of the results. Therefore, future studies are urgently needed to conduct large-sample, multi-center, prospective research to verify the universality of the conclusions and determine the optimal threshold of LINC01127 for clinical diagnosis and prognosis. Secondly, this study only detected the expression level of LINC01127 in the relatively early stage of the disease and did not systematically analyze its dynamic changes in the progression of sepsis. Further research is needed to further clarify its dynamic regulatory role and prognostic value in the host response from the time dimension. Thirdly, the mechanism research is mainly based on the single-cell model induced by lipopolysaccharide, which is difficult to fully simulate the complex cell interactions and immune heterogeneity in the human body. In the future, the original immune cells of sepsis patients and the multi-microbial animal model will be used to further verify the function and mechanism of the LINC01127/miR-34b-5p axis in vivo. Finally, this study has not clarified the direct regulatory effect of miR-34b-5p on the target gene APOB, and the analysis of the downstream pathway of LINC01127 is still not deep enough. Future research will focus on clarifying the regulatory relationship between miR-34b-5p and APOB to improve the mechanism explanation of this ceRNA regulatory axis.

In summary, our research outcomes illustrate the potential of serum LINC01127 as a novel biomarker for sepsis. LINC01127 may participate in sepsis progression by inhibiting excessive inflammatory responses and apoptosis in THP-1 cells through binding to miR-34b-5p. These findings lay the foundation for clinical diagnosis and treatment of sepsis.

## Supplementary Information

Supplementary Material 1.

Supplementary Material 2.

## Data Availability

Data can be shared upon reasonable request by the corresponding author.
