# Supplementary figures and images for: Serum LINC01127 serves as a diagnostic biomarker for sepsis and its predictive value for clinical outcomes
Source: Hereditas. 2026 Apr 6;163:61. doi: 10.1186/s41065-026-00670-1 (PMC13182082; doi:10.1186/s41065-026-00670-1)

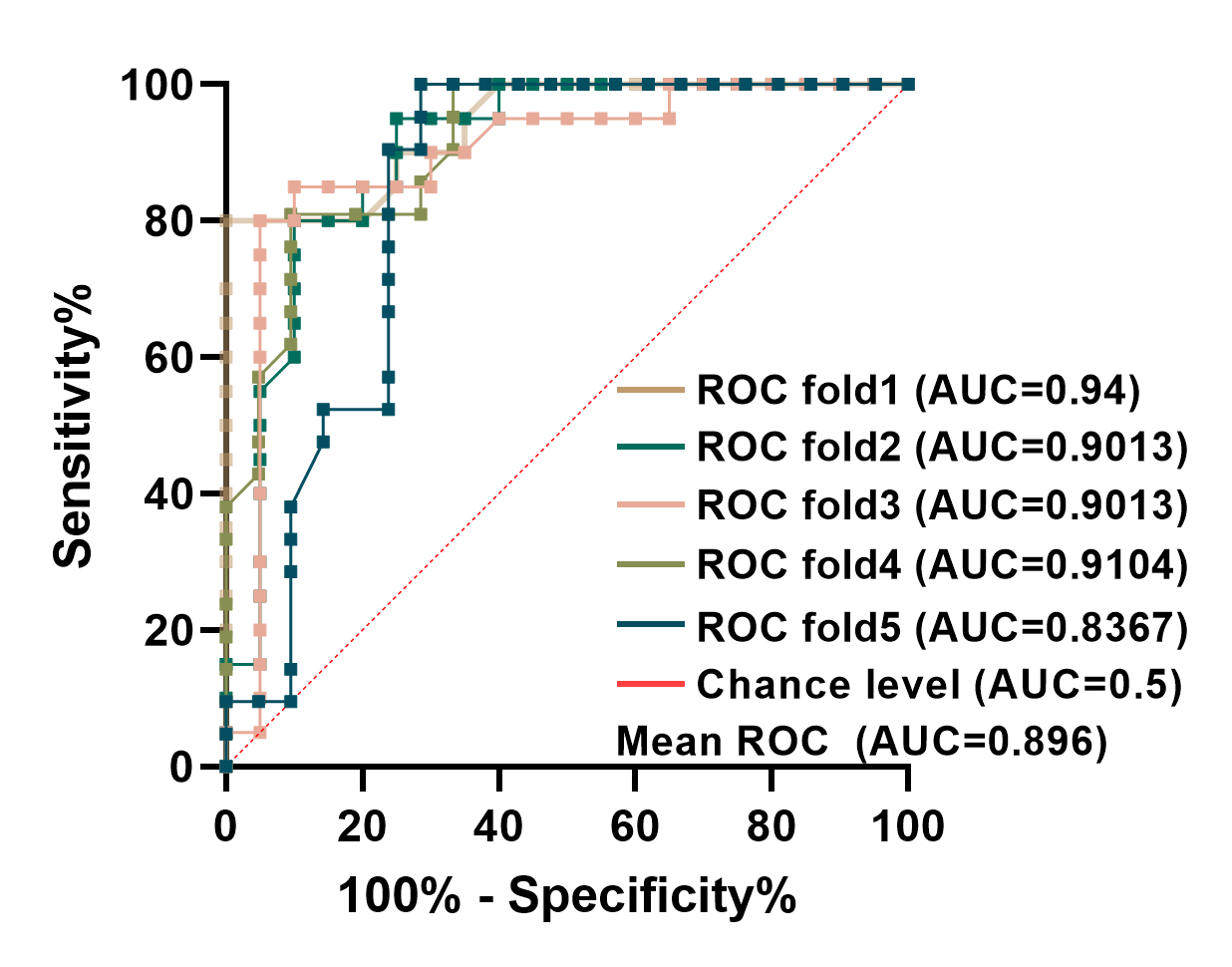

Supplement: Supplementary file 1 — Supplementary Material 1. [file 41065_2026_670_MOESM1_ESM.tif]

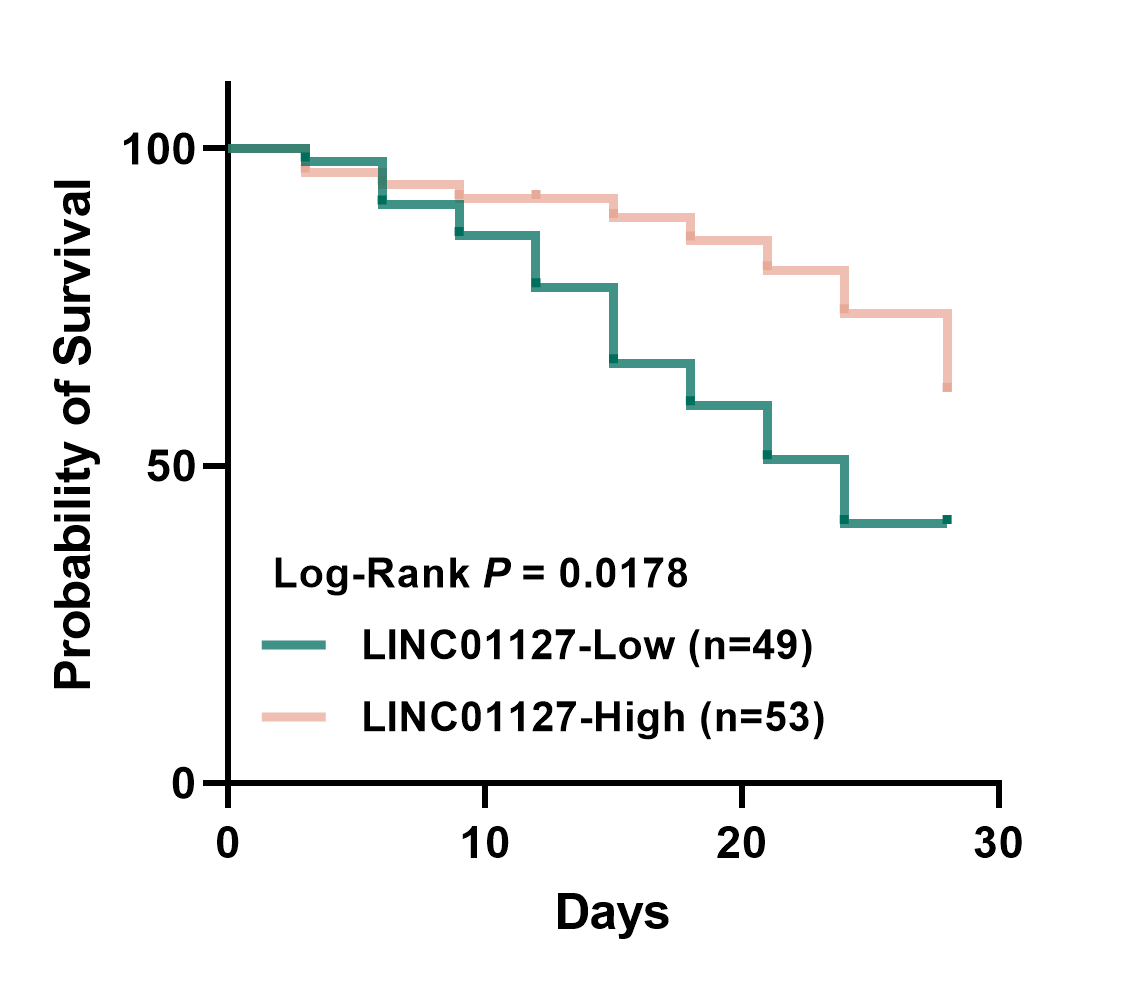

Supplement: Supplementary file 2 — Supplementary Material 2. [file 41065_2026_670_MOESM2_ESM.tif]
